# Ten Simple Rules for writing algorithmic bioinformatics conference papers

**DOI:** 10.1371/journal.pcbi.1007742

**Published:** 2020-04-02

**Authors:** Paul Medvedev

**Affiliations:** 1 Department of Computer Science and Engineering, The Pennsylvania State University, University Park, Pennsylvania, United States of America; 2 Department of Biochemistry and Molecular Biology, The Pennsylvania State University, University Park, Pennsylvania, United States of America; 3 Center for Computational Biology and Bioinformatics, The Pennsylvania State University, University Park, Pennsylvania, United States of America; Carnegie Mellon University, UNITED STATES

## Abstract

Conferences are great venues for disseminating algorithmic bioinformatics results, but they unfortunately do not offer an opportunity to make major revisions in the way that journals do. As a result, it is not possible for authors to fix mistakes that might be easily correctable but nevertheless can cause the paper to be rejected. As a reviewer, I wish that I had the opportunity to tell the authors, “Hey, you forgot to do this really important thing, without which it is hard to accept the paper, but if you could go back and fix it, you might have a great paper for the conference.” This lack of a back and forth can be especially problematic for first-time submitters or those from outside the field, e.g., biologists. In this article, I outline Ten Simple Rules to follow when writing an algorithmic bioinformatics conference paper to avoid having it rejected.

## Introduction

As a frequent program committee (PC) member of bioinformatics conferences, I sometimes find it frustrating to see a paper that potentially has a great contribution be rejected because of the way it was written. I wish that I had the opportunity to tell the authors, “Hey, you forgot to do this really important thing, without which it is hard to accept the paper, but if you could go back and fix it, you might have a great paper for the conference.” In our conference format, this type of back and forth is usually not possible. This motivated this article so that newcomers to the field have a chance to know in advance what a potential reviewer might look for in an algorithmic bioinformatics conference paper.

What do I mean by algorithmic bioinformatics conference paper? I am thinking of a subset of papers submitted to the following conferences:

• International Conference on Research in Computational Molecular Biology (RECOMB)

• Workshop on Algorithms in Bioinformatics (WABI)

• The proceedings track of Intelligent Systems for Molecular Biology (ISMB). Note that ISMB is very diverse, with different types of tracks, and this article only refers to those focusing on methods. Certain tracks may also feature papers focusing on biology, in which case some of the rules below may not apply.

• RECOMB Satellite Workshop on Massively Parallel Sequencing (RECOMB-Seq)

Other conference likely fall into this category, although I am not as personally familiar with them. The types of papers I am thinking of is the subset of papers that take an algorithm-based approach to solving a bioinformatics problem. This is largely intended to contrast with papers more rooted in statistical methodology, in which the standards are a bit different. I also focus on conference reviews, in which the process is a bit different than for a journal. When reviewing a paper for a bioinformatics journal like Oxford’s *Bioinformatics*, there is of course an opportunity for the authors to address any limitations in a revision.

I want to also add a disclaimer that this is not in any way an official statement about what PC members would look for in a review. Each conference has an official call for papers. It may sometimes state or imply necessary elements for submission, although these are typically at a fairly high level. As far as I know, there is no official policy at the level of detail presented here, and the things that each PC member looks for do not completely overlap. There is a diversity of standards and that is why each paper has multiple PC members reviewing it.

## The rules

### Rule 1: Make sure to clearly and succinctly state what the main novel contribution of the paper is

When I review, the first thing I try to identify is as follows: What is the main novel contribution of the paper? Is it an idea, a theorem, an algorithm, or a tool (i.e., software) people can use? Sometimes a paper has all these components, but not all of them contribute to the novelty of the paper. Here are some examples:

1. The paper contains an algorithm and a tool that implements the algorithm. The algorithm itself may be a simple modification of what is previously known, but the algorithm is implemented in a novel software tool for an important biological problem. If the tool performance is an improvement over previous tools, then the tool is the main contribution.

2. In another example, the main novelty is in the algorithm or in its analysis, and this is what the reader is intended to take away from the paper. The paper may have implemented a tool, but the intention of the tool is to only be a prototype to test the feasibility of the idea. The tool is not the main contribution.

3. Sometimes, the main contribution of the paper is novel biological findings without any methodological (either algorithmic or software) novelty. This is not really within the scope of an algorithmic bioinformatics conference, which has to be methodological. Certainly, having novel biological findings can serve to demonstrate the strength of the methodological contribution. But if you discover a cure for cancer by applying existing software, then it is probably outside the scope.

It is up to the authors to make the main contribution of the paper crystal clear to the reader. As a reviewer, I will then base my evaluation on what the authors claim. For example, as I will describe in Rule 8, the standards for evaluating a paper whose main contribution is a tool are different for a paper whose main contribution is a theorem. If the authors' claim is not clearly stated, then I will do my best to guess what it is. But if I make a mistake, then I may end up evaluating the paper from a completely incorrect angle.

### Rule 2: Give context within prior algorithmic work

A common scenario in which this rule is not followed is when the authors developed a method for a particular biological data set and there are no other tools designed specifically for this kind of data set or problem. However, the problem and/or solution might be very similar to what has been previously studied. For instance, many problems come down to clustering of some data points (e.g., genes in a network or reads from a sequencing experiment) or to some version of sequence alignment. The algorithmic context of such a paper is, at least in part, clustering or, respectively, alignment algorithms. Sometimes the authors provide the biological context (e.g., what is the relationship to previous approaches to finding genes in a network?) but leave out the algorithmic one (e.g., what is the relationship to previous clustering algorithms). Why is this particular problem or data set different enough so that standard clustering or alignment techniques do not apply? If the authors present a clustering algorithm for the problem but do not answer this question in the introduction, then their contribution is not placed in the algorithmic context—which makes it hard to evaluate its novelty.

### Rule 3: Make the writing clear

Some papers will contain many spelling and grammatical mistakes or ambiguous notation and terminology. These of course should be avoided, and at least spelling can be easily improved by using a spell checker. I try to do the best I can to understand the contribution of the paper, and often I do understand it in spite of these problems. In such cases, it does not greatly influence my overall decision about the paper, and I generally trust the authors to clean up the paper before publication (if it is accepted). In other cases, I cannot understand the paper after a reasonable amount of time trying. This is especially the case with ambiguous notation or terminology. In these cases, I simply cannot evaluate the paper's contribution.

### Rule 4: Do not write the paper in the style of a biology journal

In biology journals, the methods section is often written as a step-by-step manual necessary to reproduce the results (i.e., a pipeline of processing steps on the data). This type of presentation focuses on implementation details and reproducibility rather than highlighting the novelty of the algorithm. Even if the method is novel, when it is written in this style it is hard for the reader to identify and understand the novel parts. Another aspect of this is that for a biology journal, the results section comes before the methods section. Doing this for an algorithmic bioinformatics paper is not in it of itself a problem, but it usually correlates with not enough focus being given to the method.

### Rule 5: Make sure that claims in the introduction are supported by the rest of the paper

For example, the authors claim that their tool is the fastest to date for a problem, but the results section only contains a comparison against one other tool or only on a narrow type of data. In such cases, I simply ask the authors to tone down their claims. However, sometimes the claims are central to the claimed importance of the paper, in which case the lack of proper evaluation feels a bit disingenuous. Another example is the bait and switch, in which the introduction claims that the paper presents an algorithm for some interesting problem, but what ends up being evaluated in the results is an algorithm for a slightly different problem.

### Rule 6: Make sure there is either a strong theoretical contribution or an experimental evaluation

Some contributions are theoretical—a powerful idea, a way of thinking about a problem, or a theorem that can be applied by other algorithm developers. These papers require a lot of work on the modeling or theoretical side, and it can be justifiable if experimental results are either not included or limited. However, in most other cases, experimental evaluation is essential to a paper. If this is missing or is inappropriate to the problem, it can be impossible to evaluate the strength of the contribution.

### Rule 7: Compare against other work

The authors sometimes find it obvious that their method should work much better than anything else out there. They may be right, but it is important to demonstrate this in the paper by finding the most compelling alternative approach and comparing their method against it.

When doing an empirical comparison, the authors have wide leeway in choosing which data sets, computing configuration, or parameters to use. This is sometimes referred to as researcher degrees of freedom [1]. It is important that the authors are forthright about how their choices affect the evaluation. For example, while it is normal to use data sets that would demonstrate the advantages of the presented algorithm, it should nevertheless be made clear that the data sets were chosen with this in mind and that there may be other data sets on which the other tools would perform better.

### Rule 8: If the main contribution of the paper is a tool, then the software should be usable

At the very least, I should be able to download the software, install it, and run it on a toy input that is provided in the download. If I can see that the tool already has some users (e.g., through GitHub activity), then this is enough to demonstrate its usability, and I may not bother to try it out myself. On the other hand, if the paper contains a tool that is only a prototype and is not the main contribution, then the usability of the software is not something I consider important. However, I still expect it to at least be publicly available for download.

### Rule 9: Give a precise description of the algorithm, argue its correctness, and verify the correctness of the method's computations explicitly in the experiments

This rule is especially applicable when the main contribution of the paper is an algorithm. It has three distinct parts. The first is to describe the algorithm precisely. This means to explicitly state (1) what the input is and what the assumptions are made about it are, (2) what the output is, independently of what the algorithm is, and (3) what the algorithm that converts the input to the output is. These should be stated in a way that is unambiguous, using mathematical notation and/or pseudocode to the extent it facilitates preciseness.

Second, it should be argued why the algorithm achieves its stated goal. Ideally, the goal should be stated explicitly as a problem formulation [2], in which case a formal proof of correctness (or at least a coherent argument) should be given. Sometimes, the correctness is obvious from the construction, especially in the case of a data structure, and a separate proof is not needed. It should also be made clear if the algorithm solves the problem exactly or is a heuristic. If the algorithm is a heuristic and no argument of correctness is necessary, it should be made clear.

Finally, if the algorithm is evaluated empirically, it is essential that the correctness of the algorithm is explicitly verified for the experiments, if possible. This can be a simple one line that says, for example, “We verified that the new data structure gives the same answers to queries as the previous one on all the evaluated data sets.” However, without this check, how does the reader know that the algorithm is not twice as fast as the competition just because it has a bug? Sometimes, however, a verification is not possible when the ground truth is ambiguous.

### Rule 10: Give a theoretical and/or empirical analysis of running time or memory usage

In most cases, it is important for an algorithmic bioinformatics paper to present the running time and memory usage of the algorithm, either through experimental evaluation and/or theoretical analysis. This is a very natural thing to do for computer scientists, but I sometimes find that researchers with a different background forget to include this. In other cases, the authors do not include any memory or time analysis because they know that it is tiny and besides the main point, but it may not be at all obvious to the reader. In such cases, a simple statement to the effect that the memory usage or running time is negligible and/or unimportant would suffice. Finally, make sure to state the specific details of the machine used, to the extent they are relevant to your algorithm; for example, "Intel Xeon CPU with 512 GB of RAM and 64 cores at 2.10 GHz."

## Conclusion

This list includes only the most basic rules and is not intended to be exhaustive. In a competitive venue, a paper is usually accepted based on its strengths rather than a lack of weaknesses, and following these simple rules will not necessarily get your paper accepted. However, in my experience, breaking these rules can significantly decrease a paper's chance to be accepted.

These rules are also focused on aspects that are somewhat specific to algorithmic bioinformatics in relation to biology. There are of course much broader aspects about how to write a scientific paper, e.g., cohesion, conciseness, clarity, structure, and argumentation. These are outside the scope of this paper, but there are excellent books [3, 4] and even other Ten Simple Rules articles [5] that address these aspects.
